# Neuromuscular Electrical Stimulation During Hemodialysis Enhances Exercise Capacity in Patients with End-Stage Renal Disease: A Pilot Randomized Controlled Trial

**DOI:** 10.3390/jcm14217702

**Published:** 2025-10-30

**Authors:** Amal Machfer, Achraf Ammar, Halil İbrahim Ceylan, Firas Zghal, Wael Daab, Hassen Ibn Hadj Amor, Hamdi Chtourou, Raul Ioan Muntean, Mohamed Amine Bouzid

**Affiliations:** 1 Research Laboratory: Education, Motricité, Sport et Santé, EM2S, LR19JS01, High Institute of Sport and Physical Education, University of Sfax, Sfax 3000, Tunisia; amalmachfer@gmail.com (A.M.); hamdi.chtourou@isseps.usf.tn (H.C.); bouzid.mohamed-amine@hotmail.fr (M.A.B.); 2 Department of Training and Movement Science, Institute of Sport Science, Johannes Gutenberg-University Mainz, 55099 Mainz, Germany; ammar.achraf@ymail.com; 3 Research Laboratory, Molecular Bases of Human Pathology, LR19ES13, Faculty of Medicine of Sfax, University of Sfax, Sfax 3029, Tunisia; 4 Interdisciplinary Laboratory in Neurosciences, Physiology and Psychology: Physical Activity, Health and Learning (LINP2), UFR STAPS (Faculty of Sport Sciences), Paris Nanterre University, 92000 Nanterre, France; 5 High Institute of Sport and Physical Education of Sfax, University of Sfax, Sfax 3029, Tunisia; 6 Department of Physical Education of Sports Teaching, Faculty of Sports Sciences, Atatürk University, 25240 Erzurum, Türkiye; 7 Complexité, Innovations, ActivitésMotrices et Sportives (CIAMS) Université Paris-Saclay, 91405 Orsay, France; zghal.firas2012@yahoo.fr; 8 College of Sport Science, University of Kalba, Sharjah 27272, United Arab Emirates; wael.daab@ukb.ac.ae; 9 Department of Cardiology, Tahar Sfar Hospital, Mahdia 5111, Tunisia; hassenibnhadjamor@yahoo.fr; 10 Department of Physical Education and Sport, Faculty of Law and Social Sciences, University “1 Decembrie 1918” of Alba Iulia, 510009 Alba Iulia, Romania

**Keywords:** fatigue, chronic kidney disease, hemodynamic, neuromuscular electrical stimulation

## Abstract

**Background**: Exercise capacity is markedly impaired in patients with end-stage renal disease (ESRD) due to neuromuscular dysfunction and reduced oxygen delivery. This study aimed to investigate the effects of NMES during HD on exercise capacity in patients with ESRD. It specifically examined neuromuscular and hemodynamic adaptations. **Methods**: Twenty-two patients with ESRD were randomized to a neuromuscular electrical stimulation training group (NSTG, *n* = 11) or a control group (CG, *n* = 11). The NSTG underwent intradialytic quadriceps NMES three times per week for 12 weeks (40 min/session). Exercise capacity was evaluated via sustained isometric contraction at 50% of maximal voluntary contraction (MVC) until exhaustion. Neuromuscular function was assessed through voluntary activation (ΔVA) and potentiated twitch force (ΔQ_tw,pot_), while muscle oxygenation (ΔO_2_Hb, ΔHHb, ΔTHb) of the vastus lateralis was continuously monitored using near-infrared spectroscopy. **Results**: After the intervention, the NSTG showed a significant +20% increase in Tlim (103.9 ± 14.4 s to 123.3 ± 16.6 s; *p* = 0.01) and +30% improvement in MVC (421.3 ± 24.9 N to 550.4 ± 20.3 N; *p* < 0.01), while no improvements were observed in CG. Resting VA increased by ≈7% in NSTG (90.2 ± 3.7% to 96.8 ± 2.5%; *p* = 0.012). Improved muscle oxygenation and reduced twitch force suggest enhanced oxidative capacity and greater peripheral fatigue tolerance. **Conclusions**: Intradialytic NMES elicited robust improvements in exercise capacity, muscle strength, and oxygen utilization in ESRD patients by enhancing both central activation and peripheral oxidative adaptations. These findings support NMES as a feasible and effective rehabilitative strategy to counteract fatigue and functional decline in the ESRD population.

## 1. Background

Chronic kidney disease (CKD) is defined by persistent structural or functional abnormalities of the kidneys lasting at least three months, leading to significant metabolic and physiological disturbances [1,2]. These effects are especially severe in end-stage renal disease (ESRD), the most advanced form of CKD.

Beyond its effects on renal function, ESRD is associated with impairments in skeletal muscle function and exercise capacity, which are linked to reduced physical function, lower quality of life, and morbidity [3]. Etiology of these impairments is multifactorial, including muscle atrophy, inflammation, uremic toxicity, altered hemodynamics, and reduced oxygen delivery to active muscles [4]. Addressing these challenges is therefore essential, as improving exercise capacity was linked to reduced mortality and better quality of life in the ESRD population.

Rehabilitation therapies through physical exercise have been introduced to ESRD patients to reduce muscle weakness, improve exercise capacity, and decrease the risk of developing cardiovascular disease resulting from renal failure [5]. Indeed, reduced exercise capacity is strongly associated with mortality in ESRD patients. Although aerobic and resistance training are recommended for improving physical capacity in patients with ESRD, these interventions can be poorly tolerated due to frequent symptoms such as hypotension, fatigue, and cardiovascular instability during or after sessions [6]. Therefore, introducing alternative rehabilitation programs with lower clinical implications, such as neuromuscular electrical stimulation, is crucial [7].

Neuromuscular electrical stimulation (NMES) has emerged as an innovative and feasible alternative to traditional exercise in the ESRD population. By eliciting muscle contractions through electrical impulses, NMES allows for targeted activation of skeletal muscles without requiring voluntary effort. Mechanistically, NMES bypasses the central nervous system by directly depolarizing motor neurons through externally applied electrical impulses. This leads to involuntary muscle contractions, independent of patient effort. In contrast, voluntary exercise requires central activation via cortical motor pathways to initiate muscle contraction. As such, NMES can activate muscle fibers even in individuals with impaired neuromuscular function or reduced exercise tolerance. Previous studies have demonstrated that NMES improves muscle strength, endothelial function, and physical performance in various populations, including those with chronic diseases [8,9].

In ESRD patients, intradialytic rehabilitation with NMES has been explored as a strategy to counteract muscle atrophy and improve physical function. Dobsak et al. (2012) conducted an 8-week intradialytic NMES intervention in hemodialysis patients, reporting improvements in muscle strength and exercise tolerance [7]. However, their study did not assess neuromuscular activation or muscle oxygenation. In contrast, our study extends this work by incorporating mechanistic outcomes, particularly neuromuscular and hemodynamic adaptations. Such comprehensive assessments are critical for optimizing NMES protocols and extending their application to this population.

Considering the multifactorial peripheral dysfunction in ESRD, including impaired neuromuscular and oxygen delivery systems [10,11,12], there is a strong rationale for testing targeted rehabilitation strategies such as NMES. Therefore, this study aimed to investigate the effects of NMES intervention applied during HD on exercise capacity in ESRD patients, with a focus on neuromuscular and hemodynamic mechanisms. By assessing changes in neuromuscular function, muscle oxygenation, and endurance capacity, this research aims to provide a mechanistic framework for the observed benefits of NMES and to further establish its role as an accessible and effective intervention for enhancing physical fitness and health in patients with ESRD.

## 2. Methods

### 2.1. Participants

**Figure 1:**
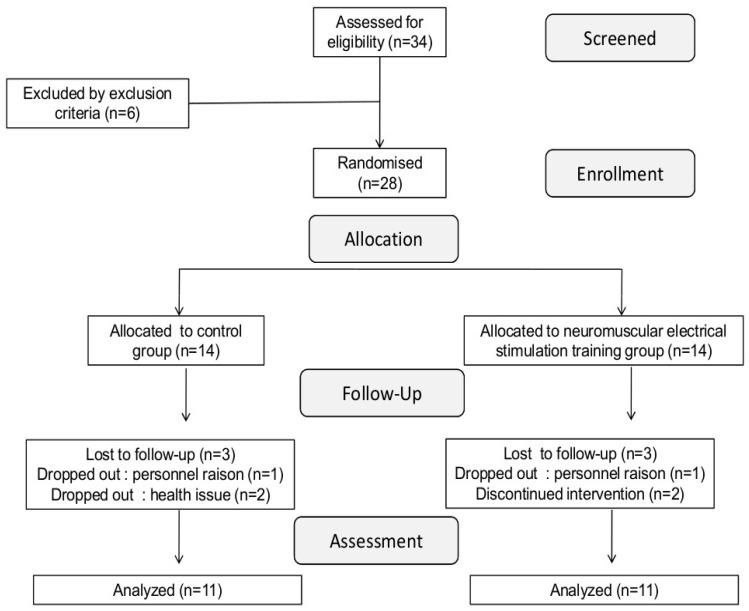
Schematic illustration of the study protocol. Neuromuscular function and muscular hemodynamic responses were assessed before and after the NMES intervention period. In each period, fatigue was assessed before and after exercise using femoral nerve stimulation (F). MVC: Maximal voluntary contraction, Tlim: time to task failure. Muscular hemodynamic responses were assessed through the exercise test by NIRS.

All participants provided written informed consent before enrollment. The study protocol received ethical approval from the Regional Research Ethics Committee (CPP SUD N° 11/2019), was registered in the Pan African Clinical Trial Registry (PACTR202206634181851), and adhered to the principles of the Declaration of Helsinki. Initially, 34 patients with end-stage renal disease (ESRD) were screened from medical records and recruited from local renal units. Eligibility criteria included undergoing chronic dialysis therapy for at least 12 months. Six patients were excluded based on predefined criteria, including cardiovascular conditions such as active coronary artery disease and recent ischemic events (*n* = 2), uncontrolled hypertension during dialysis (≥180/95 mmHg, *n* = 2), excessive interdialytic fluid accumulation (>3 L, *n* = 2), and anemia (Hb < 9.0 g/dL, *n* = 1). Among the remaining 28 eligible participants, an additional six were excluded due to contraindications related to exercise testing. Consequently, 22 patients with ESRD completed the study protocol and were included in the final analysis (Figure 1).

### 2.2. Study Design

This study employed a randomized, controlled clinical trial design. Participants were randomly assigned to either the control group (CG) or the neuromuscular electrical stimulation training group (NSTG) using block randomization with a fixed block size of 4 to ensure balanced group allocation. The randomization sequence was computer-generated (random.org) by an independent researcher not involved in recruitment or assessment. Allocation concealment was maintained using sequentially numbered, sealed, opaque envelopes that were opened only after baseline testing. Both baseline and post-intervention assessments were conducted on non-dialysis days, with the final evaluation occurring at the end of the 12-week training period.

Due to the nature of the intervention, participant blinding was not feasible. However, outcome assessors were blinded to group allocation for all performance and physiological measures, including time to task failure (Tlim), to reduce assessment bias (Table 1).

Each participant attended the laboratory on three separate occasions: one familiarization session and two experimental sessions—conducted before and after the intervention—during which they completed the fatiguing exercise protocol. 

### 2.3. Intervention Protocol

All patients received standard HD therapy; however, those in the NSTG also underwent intradialytic neuromuscular electrical stimulation (NMES) targeting the quadriceps muscles in both lower limbs. The NMES protocol was designed based on established methodological recommendations for effective muscle strengthening via surface electrical stimulation [13]. Each participant in the NSTG completed a total of 36 NMES sessions, and each NMES session consisted of 80 contractions delivered at a frequency of 50 Hz and a pulse duration of 400 μs. The duty cycle was set to 10 s of contraction followed by 20 s of rest, for a total stimulation time of 40 min per session. Two self-adhesive electrodes (5 × 5 cm) were placed on the quadriceps (vastus medialis and vastus lateralis) following anatomical landmarks. Sessions were conducted three times per week for 12 weeks during hemodialysis.

Stimulation intensity was progressively increased during the first sessions to reach the highest tolerable level without causing pain, which corresponded to a mean current of 51 ± 9 mA across participants. This value remained stable throughout the training period.

The participants were positioned with their trunks at 120° and their knees flexed to 60°, aligning with the optimal position for generating maximal muscle force (where 0° represents full leg extension) [14].

NMES contractions were elicited using a portable, programmable stimulator (Genesy 1200 PRO, Globus Italia, Codognè, Italy). Rectangular waves with extended pulse durations (300–400 µs) were chosen, as they are known to produce the most effective quadriceps contractions [15]. The stimulation frequency was set at 50 Hz, which falls within the optimal range (50–120 Hz) for strength training [16].

Neuromuscular stimulation was administered using self-adhesive bipolar electrodes (MyoTrode, 5 × 5 cm; Globus, Brescia, Italy), with four electrodes applied to each leg. The stimulation intensity was gradually increased to the highest level each participant could tolerate without discomfort, ensuring the elicitation of effective muscle contractions. Electrode placement followed a standardized anatomical protocol: two negative electrodes were positioned near the proximal insertion points of the vastus medialis (VM) and vastus lateralis (VL), approximately 1–3 cm below the inguinal ligament, over the femoral triangle. The corresponding positive electrodes were aligned as closely as possible with the motor points of the VM and VL muscles. These motor points were identified by surface probing to determine the minimal stimulation threshold [17]. Throughout each session, electrodes remained securely in place without repositioning. Electrode placement was standardized for all participants using anatomical landmarks (e.g., the midpoint between the anterior superior iliac spine and the patella for the quadriceps) and recorded on a body diagram. The same electrode position and orientation were used for each session, verified by visual inspection and participant feedback”.

Participants were advised to continue their usual daily activities and lifestyle habits throughout the study. All NMES sessions were carried out by trained personnel and closely monitored by medical professionals to ensure protocol compliance and participant safety.

## 3. Study Outcomes

### 3.1. Fatiguing Exercise Protocol

**Figure 2:**
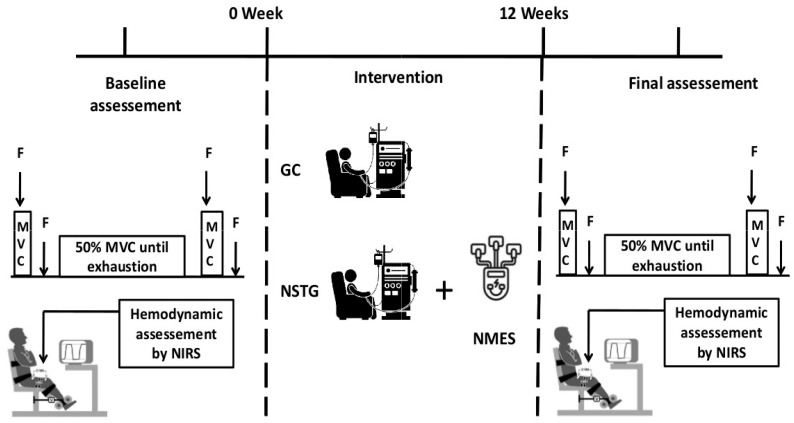
Flowchart of study participants, MVC, maximal isometric voluntary contraction.

During the experimental session, participants performed a constant-force isometric contraction at 50% of their maximal voluntary contraction (MVC) until exhaustion (Figure 2), using an isometric dynamometer (Good Strength, Metitur Ltd., Jyvaskyla, Finland) with the dominant leg. Real-time force feedback was displayed on a computer screen, with visual guidelines (±2 N) provided to help maintain target force. Endurance time (Tlim) was defined as the point at which the participant was no longer able to maintain the target force level (50% of MVC) for more than five consecutive seconds, despite strong verbal encouragement. Voluntary cessation due to discomfort or fatigue was also accepted as an endpoint if it occurred before force failure.

### 3.2. Data Collection and Analysis

#### 3.2.1. Assessment of Neuromuscular Function

Neuromuscular function was evaluated both before and immediately after the fatiguing exercise protocol. The assessment encompassed maximal voluntary contraction (MVC), surface electromyographic (EMG) activity of the quadriceps, voluntary activation (VA), and potentiated twitch force (Q_tw,pot_). Participants were positioned on an isometric dynamometer, with the dominant leg secured via a cuff connected to a strain gauge (see Figure 1). Electrical stimulation was administered to the femoral nerve using a constant-current stimulator (Digitimer Ltd., Hertfordshire, UK), delivering 1 ms square-wave pulses at voltages up to 400 V. Before each testing session, the stimulation intensity was increased incrementally until the evoked twitch force plateaued, indicating supramaximal stimulation of the femoral nerve. This procedure was conducted and confirmed for all participants to ensure consistent and valid assessments of voluntary activation and twitch force. To ensure supramaximal stimulation and minimize axonal hyperpolarization, the final stimulation intensity was set at 150% of the level required to reach the force plateau [18]. For each MVC, two femoral nerve stimulations were applied: a superimposed twitch during the MVC and a potentiated twitch (Q_tw,pot_) 3 s after contraction. Neuromuscular (∆MVC%), peripheral fatigue as the change in Q_tw,pot_ (∆_Qtw,pot_%), and central fatigue as exercise-induced changes in VA, calculated from superimposed and potentiated twitch amplitudes as follows [19]:

VA (%) = [1 − (Superimposed twitch/Q_tw,pot_)] × 100

#### 3.2.2. Electromyographic Recordings

Electromyographic (EMG) signals were recorded using bipolar silver chloride surface electrodes (Dormo Electrodes, Telic Group, Barcelona, Spain), placed lengthwise over the muscle belly in accordance with SENIAM guidelines, with an inter-electrode distance of 20 mm [20]. Signals were amplified (Octal Bio Amp ML 138, ADInstruments, New South Wales, Australia), band-pass filtered (10 Hz–1 kHz), and sampled at 2 kHz using LabChart 7 software. For each potentiated twitch (Q_tw,pot_), the peak-to-peak amplitude of the maximal muscle action potential (M-wave, Mmax) was measured. EMG activity was quantified using root-mean-square (RMS) values, calculated over a fixed 1 s window that began 500 milliseconds before the peak force output. Surface EMG signals were normalized to the maximal M-wave amplitude (RMS/Mmax) to account for inter-individual variability in signal amplitude and electrode placement. This method is widely used in neuromuscular studies because it provides a physiologically meaningful reference, reflecting the maximum compound muscle action potential. Compared to other normalization methods, RMS/Mmax allows for better comparison across sessions and subjects, especially in clinical populations where muscle size and signal quality may vary.

#### 3.2.3. Muscular Hemodynamic Measurement

Near-infrared spectroscopy (NIRS) (Oxymon Mk III; Artinis Medical Systems, Zetten, The Netherlands) was used to monitor muscle oxygenation during exercise. The NIRS device was factory-calibrated before data collection. In addition, standard pre-session checks were performed to ensure signal stability and proper skin contact. The emitter–detector pair was positioned on the belly of the right VL, midway between the greater trochanter and lateral epicondyle, and secured with adhesive tape and an elastic bandage to minimize movement artifacts. The same trained investigator positioned the probe at the same anatomical site for all sessions. Probe placement was carefully standardized by marking the skin and recording anatomical landmarks (e.g., mid-thigh) to ensure consistent positioning across sessions. The area was shaved and cleaned before each test to improve signal quality, and an elastic bandage was used to secure the probe and minimize movement. Subcutaneous adipose tissue thickness was visually assessed to ensure it did not exceed the device’s measurement depth (~2.5 cm), and participants with excessive adiposity at the measurement site were excluded.

Data were sampled at 10 Hz, and changes in oxyhemoglobin (ΔO_2_Hb) and deoxyhemoglobin (ΔHHb) were calculated using the Beer–Lambert law at 780 and 850 nm wavelengths. Total hemoglobin (ΔTHb = ΔO_2_Hb + ΔHHb) was used as an index of regional blood volume [21]. ΔHHb served as a sensitive indicator of tissue deoxygenation due to oxygen extraction [21]. NIRS-derived variables (ΔO_2_Hb, ΔHHb, ΔTHb) have demonstrated high test–retest reliability in clinical populations, with coefficients of variation typically <5%. All NIRS values were normalized to a 1-min pre-exercise baseline (set at zero µM) to express relative changes throughout the protocol.

### 3.3. Statistical Analyses

The sample size estimation was informed by prior research examining the effects of NMES training in individuals with ESRD compared to a control group [7]. Based on an anticipated effect size of 0.93, with a significance level (α) of 0.05 and statistical power (1 − β) of 0.80, a minimum of 10 participants per group was determined to be necessary to detect significant differences in maximal voluntary force across time points and between groups. This calculation was performed using G*Power (version 3.1.9.4) and was tailored for a two-way repeated-measures ANOVA design.

Statistical analyses were performed using Statistica for Windows software (version 12.0). Data were assessed for normality using the Shapiro–Wilk test and for homogeneity of variances using Levene’s test. All assumptions were met, and no data transformations were required. Although test–retest reliability was not formally calculated in this sample, MVC, VA, and NIRS measurements were performed using standardized protocols previously validated in ESRD populations.

Participant characteristics, Tlim data, and changes in fatigue from baseline to post-exercise (∆MVC%, ∆Q_tw,pot_%, and ∆VA) were compared before and after the NMES intervention period using a Two-Way ANOVA (group × intervention). A two-way ANOVA (Exercise × intervention) was used to test differences in NIRS data (O_2_Hb, HHb, and THb) across groups. For NIRS data, the time effect corresponded to relative intensity levels 10%, 20%, 30%, 40%, 50%, 60%, 70%, 80%, 90%, and 100% of Tlim. Differences in neuromuscular fatigue parameters (i.e., MVC, Q_tw,pot_, and VA) were assessed using three-way ANOVA (group x intervention x Exercise). When a significant difference was found, multiple-comparison analysis was performed with the Bonferroni post hoc test.

## 4. Results

### 4.1. Exercise Performance

Regarding exercise performance, at baseline, Tlim was similar between CG and NSTG (107.44 ± 22.03 s and 103.89 ± 14.43 s, respectively, *p* = 0.53). However, following the NMES intervention period, Tlim significantly increased in the NSTG (+20%; *p* = 0.011), but there was no significant change in CG (−6%; *p* = 0.21) (Table 2).

**Figure 3:**
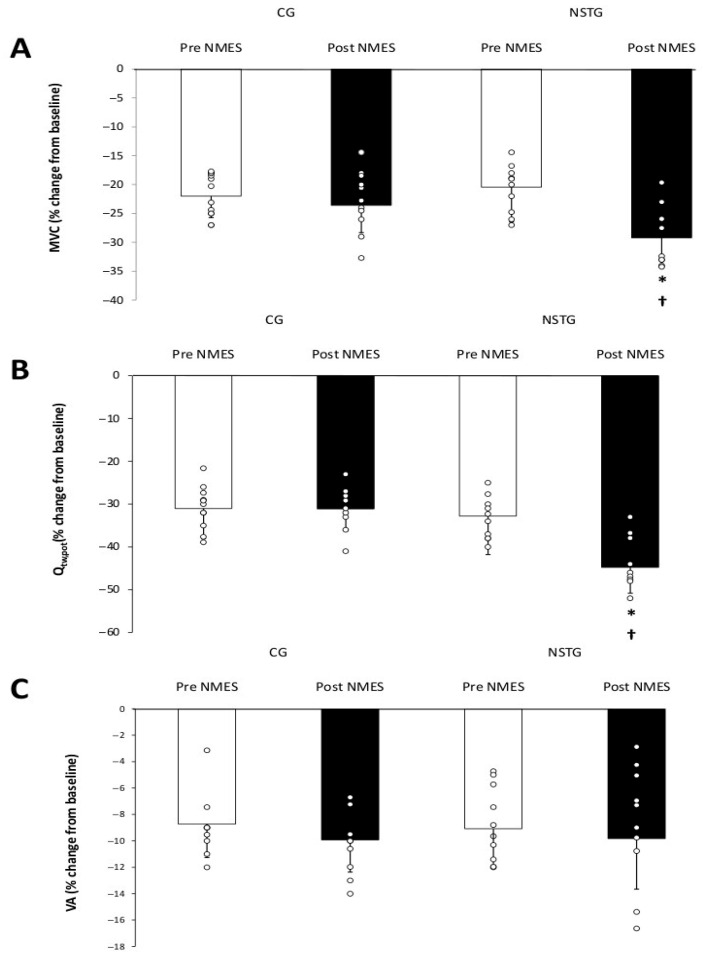
Neuromuscular fatigue after exercise performed before and following the NMES intervention in NSTG and CG participants. MVC, maximal isometric voluntary contraction; Q_tw,pot_, quadriceps potentiated twitch force; VA, voluntary activation of quadriceps motor units. (**A**): MVC, (**B**): Q_tw,pot_, (**C**): VA. * *p* < 0.05 vs. pre NMES intervention, ^†^ *p* < 0.05 vs. CG.

Concerning MVC, statistical analysis demonstrated a significant interaction effect (group*exercise*intervention) (*p* = 0.016). Post hoc analysis showed that following the NMES intervention period, resting MVC values significantly increased in the NSTG (+30%; *p* < 0.01), but with no significant changes in CG (−5%; *p* = 0.39) (Figure 3A). Moreover, ΔMVC (rest to post exercise) was significantly higher following NMES intervention compared to baseline in the NSTG (−29.1 ± 4.2% and −20.5 ± 6.1%, respectively; *p* = 0.03). No significant changes, however, were observed in ΔMVC between the pre- and post-intervention periods in CG (−21.3 ± 3.2% and −23.5 ± 4.1%, respectively; *p* = 0.61).

### 4.2. Central and Peripheral Fatigue

Neuromuscular fatigue parameters are shown in Table 2. Results revealed a significant exercise (*p* < 0.001) and group (*p* = 0.03) effects for Q_tw,pot_. Baseline and post-exercise values of Q_tw,pot_. did not change in NSTG and CG between the pre- and post-intervention periods. In addition, ΔQ_tw,pot_ was significantly higher following NMES intervention than at baseline in the NSTG (−44.1 ± 5.1% and −32.1 ± 4.4%, respectively; *p* = 0.02) (Figure 3B). These values are expressed as percentage changes from baseline.

Regarding central fatigue, statistical analysis showed a significant intervention (*p* = 0.012) and group (*p* < 0.001) effects. NMES intervention period led to a significant increase in resting VA values in NSTG (90.17 ± 3.66 vs. 96.78 ± 2.5, respectively; ≈ +7%; *p* = 0.012). However, no significant changes were observed in resting and post-exercise VA values in GC. Moreover, compared to baseline, ΔVA values did not significantly change following the NMES intervention in NETG (9.6 ± 2.7% and −9.8 ± 3.8%, respectively; *p* = 0.48) and CG (−8.7 ± 2.1% and −9.9 ± 2.4%, respectively; *p* = 0.29) (Figure 3C).

### 4.3. Electromyography

RMS/Mmax values and M wave data for VL, VM, and RF muscles are presented in Table 2. Statistical analysis showed significant effects only for the M wave (*p* < 0.05) and RMS/Mmax (*p* < 0.01). Compared with resting values, the exercise protocol led to decreases in M wave and RMS/Mmax values in both groups at baseline and after the NMES intervention period (*p* = 0.025). Moreover, no significant changes in M wave and RMS/Mmax values were observed between the pre- and post-NMES intervention periods in both groups (*p* = 0.19 and *p* = 0.56, respectively).

### 4.4. Vastus Lateralis Hemodynamic and Oxygenation

**Figure 4:**
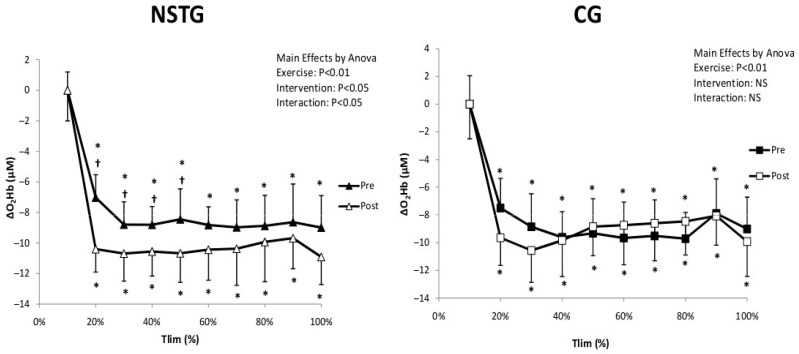
Recordings made by NIRS from the vastus lateralis change in ∆O_2_Hb in NSTG and CG before (Pre) and following (Post) the NMES intervention. ∆O_2_Hb: oxyhemoglobin. Values are means ± SD. * *p* < 0.05 vs. baseline ^†^ *p* < 0.05 vs. pre-intervention.

For muscle oxygen extraction (ΔHHb), statistical analysis showed a significant interaction (Exercise*Intervention) (*p* = 0.014) effect. ΔHHb significantly increased compared to baseline values in both groups from 20% of Tlim in both groups (*p* = 0.002). Additionally, in the NSTG, statistical analysis revealed that ΔHHb values were significantly higher between 20 and 50% of the Tlim following the NMES intervention period (*p* = 0.034). However, no significant difference was observed in the CG between pre- and post-intervention periods (*p* = 0.31) (Figure 4).

**Figure 5:**
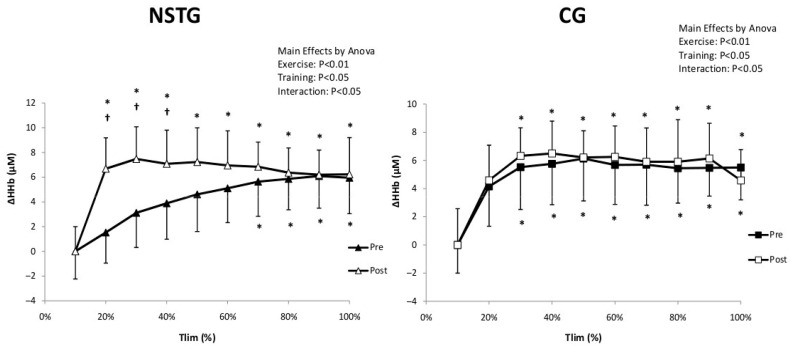
Recordings made by NIRS from the vastus lateralis change in ΔHHb in NSTG and CG before (Pre) and following (Post) the NMES intervention. ΔHHb: deoxyhemoglobin. Values are means ± SD. * *p* < 0.05 vs. baseline ^†^ *p* < 0.05 vs. pre-intervention.

Regarding muscle oxygenation (ΔO_2_Hb), statistical analysis showed a significant interaction (Exercise*Intervention) effect (*p* < 0.05). ΔO_2_Hb values significantly decreased from baseline to the end of exercise in both groups (*p* < 0.01). Following the intervention period, statistical analysis revealed that ΔO_2_Hb values were significantly higher between 20 and 50% of the Tlim in the NSTG (*p* < 0.05). In contrast, no significant difference was observed between the two periods in the CG (*p* = 0.61) (Figure 5).

**Figure 6:**
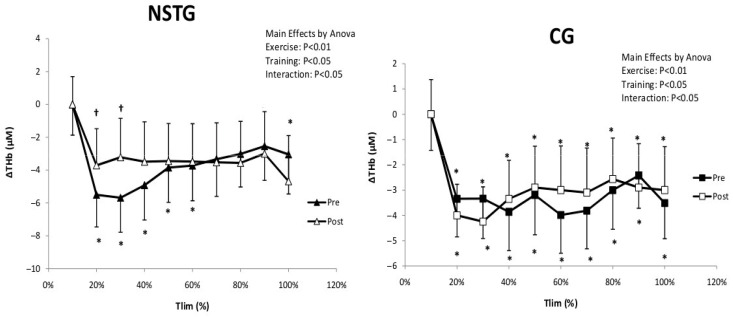
Recordings made by NIRS from the vastus lateralis change in ΔTHb in NSTG and CG before (Pre) and following (Post) the NMES intervention period. ΔTHb: total hemoglobin. Values are means ± SD. * *p* < 0.05 vs. baseline ^†^ *p* < 0.05 vs. pre-intervention.

Statistical analysis demonstrated only a significant exercise effect for muscle blood volume (ΔTHb) (*p* < 0.01). ΔTHb values significantly decreased from 20% to the end of the exercise test in both groups (*p* < 0.01). Following the intervention period, statistical analysis revealed that ΔTHb values were significantly higher between 20 and 30% of the Tlim in the NSTG (*p* < 0.05). No significant difference was observed between the two periods in the CG (*p* = 0.61) (Figure 6).

## 5. Discussion

The novelty of the current study lies in exploring the impact of intradialytic NMES on exercise capacity in patients with ESRD and in relating these results to the possible implications of neuromuscular mechanisms and muscular hemodynamic responses during physical exercise, thereby providing new mechanistic insights into the observed benefits of NMES intervention in this population. Compared with baseline, NSTG showed a 20% improvement in time to task failure (Tlim) but greater peripheral fatigue, indicating better fatigue tolerance. Significantly, this substantial improvement in exercise capacity was associated with enhanced muscle oxygen extraction and muscle oxygenation during exercise, as well as better central activation. Collectively, these findings support contributions from both central and peripheral mechanisms of NMES in the improvement of exercise capacity in patients with ESRD.

The present findings complement our earlier publication [22] based on the same patient cohort, which investigated the clinical and functional benefits of intradialytic NMES, focusing on improvements in lower-limb strength, gait performance (6MWT), balance, and mobility tests such as the TUG and STS30. That study demonstrated that NMES can improve functional capacities critical to autonomy and fall prevention in patients undergoing hemodialysis. In contrast, the current research takes a mechanistic approach to understanding how NMES induces neuromuscular adaptations. By employing near-infrared spectroscopy and electromyography, we examined muscle oxygenation and neuromuscular fatigue outcomes that were not explored in the previous publication. This physiological perspective deepens our understanding of the underlying mechanisms by which NMES improves patient outcomes, thereby reinforcing its translational value in nephrology rehabilitation programs.

### 5.1. Exercise Capacity

The increase in time to task failure (Tlim) observed in the NSTG is consistent with that reported in other clinical populations, such as patients with chronic obstructive pulmonary disease (COPD) [23] and patients with chronic heart failure [24]. In ESRD patients, reduced exercise capacity resulted from a complex interplay of factors, including chronic inflammation, impaired oxygen delivery, uremic myopathy, and cardiovascular dysfunction [25]. The NMES intervention appears to mitigate those impairments through multiple mechanisms, including improved muscle contractile efficiency and enhanced oxidative metabolism.

A possible explanation for the increased Tlim in the NSTG is that NMES-induced improvement in oxidative capacity. Indeed, in ESRD patients, uremic toxins have been reported to impair mitochondrial biogenesis and reduce oxidative enzyme activities [26]. NMES, by providing repeated muscle contractions, would stimulate mitochondrial adaptations through PGC-1α and AMPK activation [27]. These adaptations enhance skeletal muscle capacity to use oxygen during exercise, as evidenced by the improved oxygen extraction (ΔHHb) observed in our study.

While muscle fiber composition was not directly assessed in this study, the observed improvements in fatigue resistance and muscle oxygenation may be consistent with adaptations reported in previous NMES studies, which have shown transitions from type IIx to more oxidative type IIa fibers. Indeed, Gondin et al. (2011) reported that NMES promotes a fiber-type transition from IIx (fast-twitch glycolytic) to IIa (fast-twitch oxidative) fibers, which are more fatigue-resistant and better suited for sustained contractions [28]. This potential shift could partially explain the enhanced endurance capacity seen following NMES intervention

### 5.2. Neuromuscular Outcomes

The improvement in MVC highlights the dual contributions of central and peripheral adaptations to NMES in the NSTG. ESRD patients typically exhibit reduced muscle strength due to muscle atrophy, reduced voluntary activation, and peripheral neuropathy [29]. The observed increases in both MVC and VA suggest that NMES not only improves muscle contractile properties but also central motor drive.

The improvement in VA (+7%) observed in NSTG likely reflects enhanced corticospinal excitability and/or reduced supraspinal inhibition.

Repeated activation of motor neurons during NMES has been shown to improve motor unit recruitment and synchronization [14]. This mechanism is particularly relevant in ESRD patients, in whom central motor drive is often impaired by chronic inflammation and oxidative stress [30]. Moreover, increased ΔMVC and ΔQ_tw,pot_ in NSTG, further support the hypothesis that NMES enhances both central and peripheral fatigue tolerance. This is clinically significant, as better VA may translate into improved functional capacity in daily activities such as walking, rising from a chair, or climbing stairs. These activities are often impaired in patients with ESRD due to neuromuscular deficits and fatigue. Improving VA through NMES may thus support greater independence and reduce physical frailty in this vulnerable population.

In addition, peripheral adaptations could also explain MVC improvement in the NSTG. Indeed, NMES has been shown to increase muscle cross-sectional area, improve calcium handling within the sarcoplasmic reticulum, and enhance contractile protein expression [28]. These changes, taken together, would improve the muscle’s force-generating capacity and reduce fatigability in the NSTG.

Although MVC and VA improved significantly, surface EMG amplitude (RMS/Mmax) did not change. This apparent dissociation may reflect the limitations of surface EMG in detecting subtle neural adaptations, especially in clinical populations, where factors such as subcutaneous fat and electrode positioning can affect signal quality. Additionally, NMES may enhance neuromuscular efficiency or motor unit synchronization without necessarily increasing overall EMG amplitude. Thus, the functional improvements observed may be driven more by central and contractile adaptations than by gross changes in EMG activity.

### 5.3. Hemodynamic and Muscle Oxygenation

ΔHHb is often used as an index of muscle oxygen extraction, and it may also reflect local blood flow, capillary recruitment, and microvascular adaptations. The increase in ΔHHb reflects greater deoxygenation of hemoglobin during exercise, which suggests enhanced muscle oxygen extraction. Similarly, the rise in ΔO_2_Hb indicates improved delivery of oxygenated blood to the muscle. Together, these changes imply improved matching between oxygen supply and demand, potentially reflecting enhanced oxidative metabolism and vascular responsiveness in the exercising muscle.

The observed improvement in oxygen extraction (ΔHHb) highlights the role of NMES in enhancing microvascular and/or mitochondrial function. In ESRD, skeletal muscle perfusion and oxygen utilization are impaired due to reduced capillary density, endothelial dysfunction, and mitochondrial abnormalities [31,32]. Therefore, NMES may improve endothelial function through shear stress-mediated nitric oxide (NO) release, thereby enhancing capillary density and tissue perfusion [33]. In addition, the improved ΔHHb may mitigate the accumulation of metabolic byproducts, such as lactate and H^+^ ions, during exercise. This adaptation is particularly relevant for ESRD patients, who often experience early muscle fatigue due to a reduced buffering capacity and impaired lactate clearance [34].

Our findings of increased ΔHHb and ΔO_2_Hb suggest enhanced muscle oxygen extraction and improved oxidative metabolism. These results are consistent with recent studies demonstrating that NMES can stimulate mitochondrial adaptations in clinical populations. For instance, NMES has been shown to enhance oxidative enzyme activity in healthy elderly [35] and increase mitochondrial signaling pathways in adults with spinal cord injury [36]. Such adaptations likely improve endurance and energy metabolism during exercise.

The observed increases in ΔHHb suggest enhanced muscle oxygen extraction and improved local microvascular function, potentially reflecting better endothelial responsiveness. In patients with ESRD—who are at significantly elevated cardiovascular risk—even modest improvements in endothelial function have been associated with reduced arterial stiffness and improved hemodynamic profiles. Therefore, intradialytic NMES may offer not only neuromuscular benefits but also potential protective effects on cardiovascular health, warranting further investigation.

The increase in ΔTHb observed specifically at 20–30% of Tlim may reflect transient hyperemia or early vasodilatory responses during the initial phase of the task. At this stage, increased perfusion could outpace oxygen extraction, leading to elevated total hemoglobin signals. Alternatively, small fluctuations in probe positioning or muscle contraction depth could contribute to variability in optical path length, potentially affecting signal interpretation. However, since this pattern was consistent across participants, it likely reflects a physiological response rather than a measurement artifact.

Moreover, ΔTHb did not change significantly in the NSTG, suggesting that NMES may not substantially affect muscle blood volume. Therefore, the enhanced oxygen extraction (ΔHHb) would be likely related to increased mitochondrial oxidative capacity rather than changes in muscle blood volume. This finding aligns with a previous study by Ktagari et al. (2024), which demonstrated that NMES enhances skeletal muscle oxidative capacity without necessarily increasing blood flow [37].

### 5.4. Methodological Limitations

Despite its strengths, this study has several limitations. First, the sample size was relatively small, which may limit the generalizability of the findings. The study protocol includes several tests that many ESRD patients refused to undergo. Second, the mechanisms underlying NMES-induced improvements in oxygen extraction and utilization were inferred from indirect NIRS measures. Direct assessments using muscle biopsies would provide deeper mechanistic insights. Third, because the NMES intervention is visible and physical, participant blinding was not feasible. This lack of blinding is acknowledged as an inherent methodological limitation that could introduce performance bias. However, outcome assessors were blinded to group allocation to minimize detection bias. Fourth, the sample size calculation was based on changes in MVC, which was our primary outcome. Although improvements were also observed in other neuromuscular and hemodynamic variables, the study was not formally powered for each secondary endpoint. Therefore, these findings should be interpreted cautiously, particularly in light of multiple comparisons, which may increase the risk of Type I errors. Additionally, the exclusion criteria—while necessary to ensure patient safety—resulted in a relatively homogeneous and medically stable cohort. This may limit the generalizability of our findings to the broader ESRD population, especially those with cardiovascular instability or significant comorbidities. Finally, the long-term sustainability of the observed benefits remains unknown. Future studies should investigate whether continuous NMES training leads to cumulative improvements or whether periodic retraining is necessary.

## 6. Conclusions

This study demonstrated that intradialytic NMES improved exercise capacity, neuromuscular function, and oxygen utilization, suggesting it is a promising rehabilitative approach in ESRD. Future investigations should explore long-term physiological adaptations, optimal NMES protocols, and functional implications in daily activities to support NMES as a standard rehabilitative approach in ESRD care.

## Figures and Tables

**Table 1:** Descriptive data of the participants (mean ± SD): BMI, body mass index; eGFR, estimated glomerular filtration rate; Hb, hemoglobin; and Kt/V, dialysis efficiency, NS: not significant.

	NSTG Group	CG Group	*p*
Anthropometric data			
Total *n*	11	11	NS
Age (years)	37.9 ± 1.8	39.2 ± 4.1	NS
Weight (Kg)	74. 8 ± 2.3	75.9 ± 6.3	NS
Height (m)	1.7 ± 0.1	1.7 ± 0.2	NS
BMI (%)	25.1 ± 2.2	25.3 ± 3.5	NS
Physical activity score	3.8 ± 2.1	4.2 ± 2.4	NS
Comorbidities			
Diabetes mellitus type 2 (%)	3 (27%)	2 (18%)	
Hypertension (%)	2 (18%)	1 (9%)	
Clinical parameters			
Time on dialysis (months)	49.2 ± 6.8	54.8 ± 9.2	NS
eGFR (mL/min/1.73 m^2^)	9.3 ± 2.2	8.9 ± 2.7	NS
Hb (g/dL)	110.9 ± 8.8	9.9 ± 2.7	NS
Blood pressure (systolic) (mmHg)	151.6 ± 12.3	141.6 ± 10.9	NS
Blood pressure (diasystolic) (mmHg)	91.2 ± 4.5	99.5 ± 6.2	NS
Heart rate at rest (bpm)	79.2 ± 8.8	72.9 ± 6.3	NS
Kt/V	1.99 ± 0.55	1.66 ± 0.85	NS
Charlson Comorbidity Index (score)	3	3	NS

**Table 2:** Neuromuscular characteristics in control (CTR) and end-stage renal disease (ESRD) participants.

	CG				NSTG				Effects
	Pre-Intervention		Post-Intervention		Pre-Intervention		Post-Intervention		
	Rest	Post	Rest	Post	Rest	Post	Rest	Post	
MVC (N)	379.90 ± 31.51	295.50 ± 22.55 *	398.30 ± 19.02	306.50 ± 19.15 *	421.27 ± 24.90	359.73 ± 16.24 *	550.36 ± 20.33 ^†#^	388.45 ± 17.05 *	Interaction *p* < 0.001 Group *p* < 0.001 Intervention *p* = 0.02 Exercise *p* < 0.001
Q_tw,pot_ (N)	138.11 ± 4.54	96.44 ± 6.77 *	129.89 ± 3.75	86.89 ± 9.38 *	141.00 ± 4.31	94.20 ± 2.95 *	143.60 ± 7.33 ^#^	81.90 ± 3.89 *^†^	Interaction *p* = 0.39 Group *p* = 0.03 Intervention *p* = 0.07 Exercise *p* < 0.001
VA (%)	91.95 ± 1.98	85.85 ± 4.21 *	89.73 ± 2.63	80.60 ± 4.97 *	90.17 ± 3.66	84.71 ± 4.06 *	96.78 ± 2.50 ^†^	83.42 ± 6.60 *	Interaction *p* = 0.22 Group *p* = 0.18 Intervention *p* = 0.012 Exercise *p* < 0.001
VM Max (mV)	6.52 ± 1.04	5.50 ± 0.8 *	6.23 ± 1.05	5.22 ± 0.72 *	6.75 ± 0.91	5.95 ± 0.22 *	6.48 ± 0.90	6.32 ± 0.28 ^#^	Interaction *p* = 0.22 Group *p* =0.21 Intervention *p* = 0.68 Exercise *p* = 0.04
VM Max (mV)	5.53 ± 0.97	5.64 ± 0.95	5.83 ± 0.33	5.13 ± 0.64 *	5.20 ± 0.03	5.81 ± 0.55	5.47 ± 1.30	5.67 ± 0.69	Interaction *p* = 0.86 Group *p* = 0.35 Intervention *p* = 0.56 Exercise *p* < 0.001
VM Max (mV)	6.33 ± 0.97	6.44 ± 0.95	6.23 ± 1.33	6.13 ± 0.64	5.86 ± 0.52 ^#^	6.53 ± 1.39	6.14 ± 0.24	6.18 ± 1.52	Interaction *p* = 0.48 Group *p* = 0.04 Intervention *p* = 0.44 Exercise *p* = 0.99
RMS/Mmax (mV)	0.11 ± 0.01	0.08 ± 0.01 *	0.12 ± 0.01	0.09 ± 0.01 *	0.10 ± 0.01	0.08 ± 0.01 *	0.11 ± 0.01 *	0.07 ± 0.01 *	Interaction *p* =0.35 Group *p* = 0.33 Intervention *p* = 0.19 Exercise *p* = 0.01
Tlim	107.44 ± 22.03	-	99.50 ± 19.6	-	103.89± 14.43	-	123.33 ± 16.60 ^†#^	-	Interaction *p* = 0.02 Group *p* = 0.03 Intervention *p* < 0.01

Note: MVC, maximal isometric voluntary contraction; Q_tw_, quadriceps potentiated twitch force; VA, voluntary activation of quadriceps motor units; VL, vastus lateralis; VM, vastus medialis; Tlim, time to task failure. * *p* < 0.05 vs. baseline ^†^ *p* < 0.05 vs. pre-intervention ^#^ *p* < 0.05 vs. control.

## Data Availability

The data may be shared upon reasonable request to the corresponding author if the request is accepted by the Regional Research Committee for Medical and Health Research Ethics and the local Data Protection Official.
